# Immersive Virtual Reality for Treatment of Unilateral Spatial Neglect via Eye-Tracking Biofeedback: RCT Protocol and Usability Testing

**DOI:** 10.3390/brainsci14030283

**Published:** 2024-03-15

**Authors:** Alex Martino Cinnera, Valeria Verna, Matteo Marucci, Aurora Tavernese, Luisa Magnotti, Alessandro Matano, Chiara D’Acunto, Stefano Paolucci, Giovanni Morone, Viviana Betti, Marco Tramontano

**Affiliations:** 1 IRCCS Santa Lucia Foundation, 00179 Rome, Italy; a.martino@hsantalucia.it (A.M.C.); v.verna@hsantalucia.it (V.V.); a.tavernese@hsantalucia.it (A.T.); l.magnotti@hsantalucia.it (L.M.); a.matano@hsantalucia.it (A.M.); s.paolucci@hsantalucia.it (S.P.); viviana.betti@uniroma1.it (V.B.); 2 Braintrends Limited, Applied Neuroscience, 00192 Rome, Italy; matteo.marucci@uniroma1.it; 3 Department of Psychology, Sapienza University of Rome, 00185 Rome, Italy; chiaradac123@gmail.com; 4 Department of Life, Health and Environmental Sciences, University of L’Aquila, 67100 L’Aquila, Italy; 5 Department of Biomedical and Neuromotor Sciences (DIBINEM), Alma Mater University of Bologna, 40138 Bologna, Italy; marco.tramontano@unibo.it; 6 Unit of Occupational Medicine, IRCCS Azienda Ospedaliero-Universitaria di Bologna, 40138 Bologna, Italy

**Keywords:** visual deficits, eye movements, stroke rehabilitation, exergaming, neurorehabilitation, neglect

## Abstract

About one-third of stroke survivors present unilateral spatial neglect (USN) that negatively impacts the rehabilitation outcome. We reported the study protocol and usability results of an eye-tracking (ET) biofeedback immersive virtual reality (iVR) protocol. Healthy controls and stroke patients with and without USN underwent a single session of the three iVR tasks. The system usability scale (SUS), adverse events (AEs), and ET data were collected and analyzed via parametric analysis. Twelve healthy controls (six young adults and six older adults) and seven patients with a diagnosis of single ischemic stroke (four without USN and three with confirmed diagnosis of USN) completed the usability investigation. SUS results showed good acceptability of the system for healthy controls and stroke patients without USN. ET results showed a lower performance for patients with USN concerning healthy controls and stroke patients without USN, in particular in the exploration of the left visual field. The results showed that the proposed iVR-ET biofeedback protocol is a safe and well-tolerated technique in patients with USN. The real-time feedback can induce a performance response supporting its investigation such as a treatment approach.

## 1. Introduction

Stroke is a major cause of disability worldwide [1], and people may experience long-term severe disabilities [2]. Approximately 30% of stroke survivors present with unilateral spatial neglect (USN) as a result of cerebrovascular accidents [3]. Specifically, it can occur following lesions to the parietal, frontal, and/or subcortical brain areas of the right hemisphere (40%) or left hemisphere (20%) [4].

USN is characterized by a deficit in spatial attention towards stimuli on the contralesional side of the body, along with associated cognitive and motor impairments [5,6]. Altered attention and cognition towards the hemiparetic side of the body may negatively impact rehabilitation outcomes, increasing the risk of falls and prolonging hospital stays [7]. Rehabilitation approaches for USN can be classified into “top-down” methods (e.g., Visual Scanning Training) and “bottom-up” methods (e.g., Prismatic Adaptation). While the former relies on voluntary effort by the patient following the instructions of the therapists, the latter involves the manipulation of the patient’s sensory environment [8]. Although evidence in the literature supports the validity of both approaches, further studies are needed to verify their long-term effects [9].

Over the past decade, advancements in technology have opened up new opportunities for USN rehabilitation. Non-invasive brain stimulation (e.g., transcranial magnetic stimulation and transcranial electrical stimulation), pharmacological interventions (e.g., dopaminergic and noradrenergic drugs), prism adaptation, and virtual reality technologies have emerged as promising avenues for the development of effective training techniques for USN [10]. Virtual reality seems to offer a great opportunity for the rehabilitation of cognitive deficits, including the USN [11]. Indeed, recent evidence suggests that people suffering from USN can benefit from using immersive virtual reality (iVR), applied through a head-mounted display (HMD). Improvements in spatial attention and head rotation counts have been reported, particularly when patients are exposed to dynamic and/or ecological stimuli [12,13,14,15].

For evaluating USN, combining iVR with ET technology, which tracks eye movements, allows for the assessment of spatial attention [16]. This integration of ET has demonstrated significant utility in the instrumental assessment and diagnosis of USN, exhibiting comparable or superior reliability compared to conventional clinical tools [17,18,19]. Furthermore, by incorporating ET into HMDs, eye movements can serve as eye-gaze biofeedback to enhance visuospatial attention [20]. Specifically, in cases of USN where visual attention is crucial, ET biofeedback can facilitate recovery. Indeed, ET proves to be a useful approach, particularly when attentional focus plays a significant role [21]. We hypothesize that adding ET biofeedback to the iVR protocol provides a real-time top-down stimulation that may improve spatial attention deficit in USN. We propose a study protocol based on three specifically designed iVR tasks with ET biofeedback for the treatment of spatial attention in patients with USN. Moreover, in the present study, we reported the usability test and eye-gaze data results during static, dynamic, and ecological visual exploration in stroke patients and healthy controls.

## 2. Study Protocol

### 2.1. Participants

Participants were recruited through the inpatient units of the IRCCS Santa Lucia Foundation Hospital. All patients with cortico-subcortical ischemic or hemorrhagic stroke that meet the following criteria were included in the study: (i) single stroke event in the subacute phase (<180 days); (ii) diagnosis of USN; (iii) age between 18 and 80 years; (iv) without severe cognitive impairment (Mini Mental State Examination test > 23); and (v) able to maintain the sitting position. We excluded patients with (i) multiple brain lesions; (ii) severe visual deficits; (iii) neurological comorbidities; or (iv) history of epilepsy. Power analysis based on processing time results of Choi and colleagues (2021) indicated 40 patients to observe the effects (20 for group) (α = 0.05; post hoc power 80%) [22].

### 2.2. Ethical Approval

All procedures were approved by the ethical committee of the IRCCS Santa Lucia Foundation Hospital (protocol CE/2023_041). The protocol was registered in the database of the National Library of Medicine (clinicaltrials.gov, accessed on 11 March 2024, ID NCT06264713). The study followed the ethical principles for medical research on humans of the Declaration of Helsinki, and each participant signed an informed consent before undergoing the experimental procedures.

### 2.3. Protocol

A randomized triple-blind controlled clinical trial (patients, caregivers, and evaluators) was designed. All included patients were randomly assigned to the experimental group (iVR) or control group (Sham-iVR). The allocation was performed by a computer-generated randomization list and managed by a researcher (A.T.) not involved in the evaluation process. Each patient underwent nine sessions of 20 min with three sessions/week for a total of three weeks of treatment [15]. The intensity, frequency, and duration of experimental procedures overlapped in the two groups, and the treatment was applied in addition to usual care. Each session was supervised by a trained technician.

In the iVR group, in all nine sessions, the stimuli was provided in the entire visual field with both left and right directions for the moving stimuli.

In the Sham-iVR group, for the first session (session 1) and the last session (session 9), the same protocol as that of the iVR group was followed. In the other seven sessions, for the sham-iVR group, changes were made to the areas of the visual field in which the stimuli were presented. Specifically, the stimuli were provided in the central and right part of the visual field, independently from the characteristic of stimuli (static or dynamic).

To be carried out, all exercises require the use of fixation of the gaze on a target stimulus, static or moving.

To perform the iVR training, we used a head-mounted display (HMD) (Meta Quest Pro. ©2023 Meta, Meta Platforms Technologies Ireland Limited, Menars street 6, 75002, Paris, France) connected to a computer laptop running the rehabilitation software Thera (v. 2.3.8, www.thera.myndek.com, accessed on 11 March 2024). The scenarios were developed by a company specializing in VR software (Myndek s.r.l.) based on the indications provided by experts in the field of USN rehabilitation involved in the study (L.M., A.M., V.V., and A.T.) according to the literature and rehabilitation guidelines.

The ET system is embedded inside the Quest Pro headset, the calibration is performed directly inside a default VR environment provided by Meta, and interpupillary distance can be adjusted using a wheel between the lenses. Using this system in Unity, we created an invisible ray (Physics.Raycast) and monitored in real time whether the cube or other interactable elements were hit by the ray generating in the direction of the gaze. For this reason, only a gaze inside the interactable element would register as a hit or success.

### 2.4. Gazing at a Static Target (Task 1)

**Figure 1:**
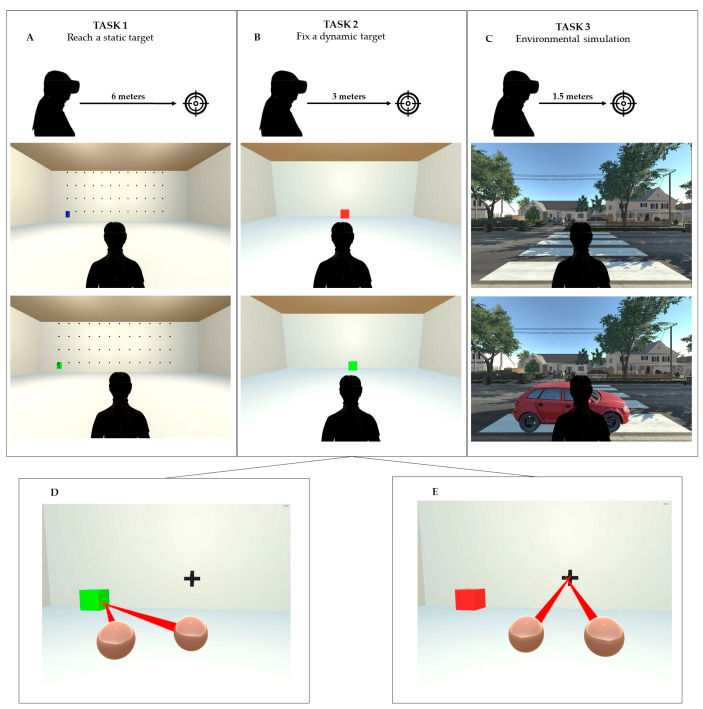
iVR tasks and ET dynamic. In the iVR scenario of Task 1 (Panel **A**), the patient observed a static task (blue); the gaze was maintained for 2 s, then the target changed color (green). In Task 2 (Panel **B**), the patient followed a moving object (red cube); if the eye’s trajectory correctly followed the target, then the cube changed color (green). In Task 3 (Panel **C**), the patient was immersed in an ecological setting reproducing a pedestrian crossing. Patients were asked to follow a red car; if the eyes correctly followed the trajectory, then the car changed color (green). The distance between eyes’ subjects and targets was 6, 3, and 1.5 m for the three tasks. Examples of correct and incorrect ET fixation for Task 2 are reported in (Panels **D**,**E**), respectively.

Once the participants had put on the HMD, they were immersed in a 360° virtual room composed of four walls. In front of the walls, 48 black bullet points (12 columns × 4 lines), distributed 1 m away from each other, were presented, covering the field of view from the extreme left (−6 m) to the extreme right (+6 m) (Figure 1A). The patient was placed 6 m in front of the wall; on a black point target, a blue target with a number from 1 to 9 appeared. The patient had to search for the dot in the space and stare at it for 2 s until it turned green. The target was shown for 6 s; if the subject was unable to fixate on the stimulus steadily and consecutively for 2 s, a new stimulus was presented.

### 2.5. Gazing at a Dynamic Target (Task 2)

Once the participants had put on the HMD, they were immersed in a 360° virtual room composed of four walls. In front of one of the walls, a cube was presented, moving at a speed of 0.4 m/s across the floor from the left (−2 m) to the right (+2 m), covering a total distance of 4 m. The patient was positioned 3 m in front of the cube and must look at it and follow its movement. If the patient correctly fixated on the target, the cube turned green. The patient was instructed to keep the cube green for as long as possible. This exercise was designed based on ideas found in the literature, which suggest a stronger response to moving stimuli (from right to left) in patients with USN compared to stationary ones. The exercise aims to simulate the pursuit of an object or subject (such as a person) moving in front of the visual field.

### 2.6. Environmental Simulation (Task 3)

In the third exercise, the participant was immersed in a 360° virtual city landscape depicting a road. A stimulus, represented by a car, traversed the street from left to right or right to left, reaching the center of the pedestrian crossing, covering a total distance of 51 m. The patient was positioned on the sidewalk, 1.5 m in front of the pedestrian crossing. They were instructed to track the car’s movement across the road until it reached the designated target area (pedestrian crossing). Positioned at the center of the pedestrian crossing, there was a red rectangle indicating the point where the vehicles disappear. The environment included realistic ecological sounds associated with car movement (Figure 1C). The objective of this exercise is to concentrate attention on reaching and monitoring a moving object or subject situated at the periphery of the visual field, within a virtual environment replicating real-life conditions (a city street). Specifically, this task, mimicking a pedestrian crossing scenario, was chosen to emphasize the importance of attending to moving stimuli, such as vehicles approaching from both sides, which is crucial in daily life situations.

### 2.7. Assessment

Each participant was evaluated at baseline (t0), at the end of the training (t1), and at the 1-month follow-up (t2). The primary outcome is the Behavioral Inattention Test (BIT). The BIT is a short screening battery of tests designed to assess the presence and interference of visual inattention during the performance of activities of daily living [23]. The secondary outcomes are: (i) Albert’s Test for the assessment of USN [24]; (ii) the Wundt–Jastrow area illusion test for the assessment of USN in the far and near extra-personal space [25]: (iii) the Kessler Foundation Neglect Assessment Process (KF-NAP) for the assessment of USN symptoms during activities of daily living [26]; (iv) copying drawings, with and without programming elements, for the assessment of constructive apraxia [27]; (v) the Stroke-Specific Quality of Life Scale (SSQoL) for the assessment of health-related quality of life after stroke [28]; and (vi) the Fugl–Meyer Assessment scale (FMA) for the assessment of sensorimotor function of the upper and lower extremities [29]. Finally, data on eye movements during exercises were collected in the first and last iVR sections (sessions 1 and 9). In task 1, we recorded the time taken to reach the stimulus with the gaze (reaction time), counting the number of stimuli stably fixated on for at least 2 s. In tasks 2 and 3, we recorded the total fixation time along the target trajectory (fixation time).

### 2.8. Expected Adverse Events and Management

No serious adverse events (AEs) have been reported in the literature, and none were expected in this study. Slight AEs, such as nausea, dizziness, disorientation, postural instability, and fatigue, have been reported in the literature [30]. Any AEs were communicated to the medical doctor who supervised the study (S.P.) and to the principal investigator (V.V.), who managed the case. Every adverse event was recorded and reported in the dissemination of the study.

### 2.9. Statistical Analysis Plan

The data distribution was analyzed using the Kolmogorov–Smirnov test. Normally distributed data were analyzed using 2 × 3 mixed model ANOVA considering the variables group (patients, controls) and time as repeated factors (3 levels). The significant time × group interactions were investigated using the Bonferroni correction post hoc test. In contrast, for non-normally distributed data, non-parametric equivalent ANOVA (Friedman test) was used. Significant results were investigated using the Mann–Whitney U test comparing the effectiveness [(ΔT/score scale) × 100)] pre/post, pre/follow-up, and post/follow-up between the two study groups.

## 3. Usability Testing

Before the start of the trial, we conducted usability testing and collected eye-tracking (ET) results from a single session of the iVR protocol. To assess perceived usability, we utilized the System Usability Scale (SUS) [31], a standardized questionnaire comprising 10 items with five response options ranging from “strongly agree” to “strongly disagree”. The SUS score provides insight into the system’s usability by evaluating the acceptability range of the system interface. Regarding ET data, in Task 1, we recorded the time taken from stimulus presentation to fixate on it. Each stimulus was presented for a maximum of 6 s. In Task 2, we recorded the total fixation time on the dynamic stimulus (a cube), expressed as a percentage of the total time. In Task 3, we recorded the fixation time from the start to the end of the car’s movement, with fixation time expressed in milliseconds. Additionally, in Task 1, we segmented the performance in the fixation time in the visual field (VF) into left, center, and right.

### Statistical Analysis

SUS scores were computed using an online Analysis Toolkit [32]. The SUS results were interpreted according to Bangor and colleagues (2008) into three classes of accessibility range. If the SUS scale has a value of 50, the usability of the system is classified as “not acceptable”. If the value is between 50 and 70, the system is considered to have “marginal” usability. If the value exceeds 70, the usability is classified as “acceptable” [33].

SUS and ET data were analyzed using one-way analysis of variance (one-way ANOVA) considering group factors like variables [young adults, older adults, stroke patients without USN (USN−), and stroke patients with USN (USN+)]. For Task 1, we used a 3 × 4 mixed model ANOVA considering the VF factor (left; center, right) and group factor (young adults, older adults, USN−, USN+). All significant interactions were further investigated via Bonferroni correction. A multiple linear correlation matrix was utilized to investigate the weight of demographic characteristics (age and years of study) on system usability and ET data. The Pearson coefficient was interpreted as follows: from 0 to 0.19 very weak, from 0.2 to 0.39 weak, from 0.4 to 0.59 moderate, from 0.6 to 0.79 strong, and from 0.8 to 1 very strong.

## 4. Results

Nineteen participants, seven stroke patients (with and without neglect) and a convenience sample of twelve healthy participants (young adults < 30 years and older adults > 30 years), underwent a single iVR session, completing the three tasks and responding to the SUS questionnaire (complete demographic characteristics are reported in Table 1). The USN patients were selected by responding to the inclusion/exclusion criteria reported in Section 2.1. Specifically, the USN+ group was composed of three patients with right cortical–subcortical frontotemporal ischemic stroke in the sub-acute phase with a confirmed diagnosis of USN assessed by a formed neuropsychologist.

### 4.1. AEs and Usability Results

**Figure 2:**
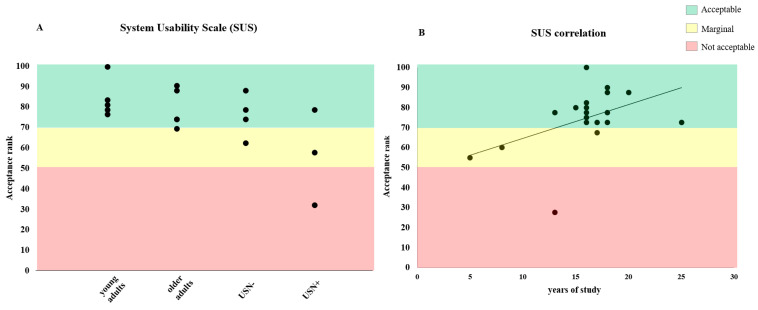
SUS results. The usability scores showed an excellent acceptance for both young and older adults and USN− (Panel **A**). In the USN+, patients recorded a lower acceptance score, probably due to the difficulty encountered in the iVR tasks. Positive significant correlation between SUS results and years of study was found (r = 0.47, *p* = 0.04) (Panel **B**), indicating that compliance with iVR is related to years of study.

No adverse events (AEs) were reported. A patient from the USN+ group reported that neck stiffness resolved without drug intervention a few minutes after a training session. The same patient reported a lower score on the System Usability Scale (SUS). Regardless of the groups, other participants and patients reported an SUS score higher than 50 points, indicating marginal acceptance. The mean SUS score for young and older adults and USN− patients was higher than 70 points, indicating good usability of the immersive virtual reality (iVR) system (see Figure 2A). However, no statistically significant difference was found between the four groups [F(3) = 1.33, *p* = 0.4] (see Table 2).

### 4.2. Correlation Results

A significant correlation was observed between the years of study and the SUS results (r = 0.47, *p* = 0.04), likely indicating that the compliance with iVR protocol is correlated with the years of study (Figure 2B). Moreover, a strong significant correlation was found between years of study and fixation time of Task 2 (r = 0.69, *p* = 0.001). No other significant correlations were found between demographic characteristics and clinical data.

### 4.3. Eye-Tracking Results

In Task 1, a statistically significant difference in the reaction time intercurrent from presentation to stimuli between the four groups [F(3) = 5.43, *p* < 0.001] was observed. The post hoc analysis showed an increased reaction time in the USN+ group with respect to the USN− and healthy controls (*p* < 0.001 with respect to each group) (all results are available in Table 2).

**Figure 3:**
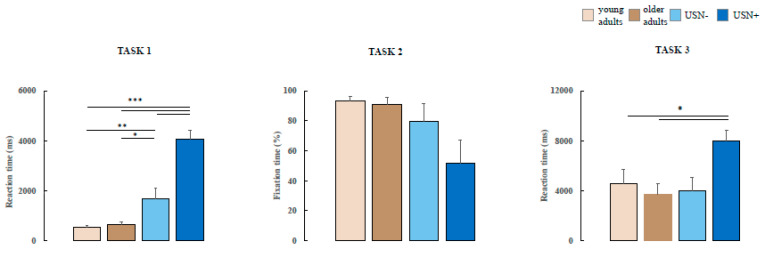
Differences in the reaction time in Task 1 across the two stroke groups (with and without unilateral spatial neglect) and healthy controls (young and older adults). Statistically significant differences were recorded in the ET results between USN+ with respect to healthy controls and USN− in the left visual fields (VF). * *p* < 0.05; ** *p* < 0.01; *** *p* < 0.001.

One-way ANOVA performed on Task 2 showed no statistical differences across the groups [F(3) = 2.24, *p* = 0.2]. In contrast, in Task 3, statistical differences across the groups were found [F(3) = 5.39, *p* = 0.03]. Post hoc analysis revealed a significantly increased in the time spent to research the stimuli between USN+ and young and older adults (*p* = 0.05 and *p* = 0.03, respectively). No differences were observed between healthy control groups and patient groups (see Figure 3).

**Figure 4:**
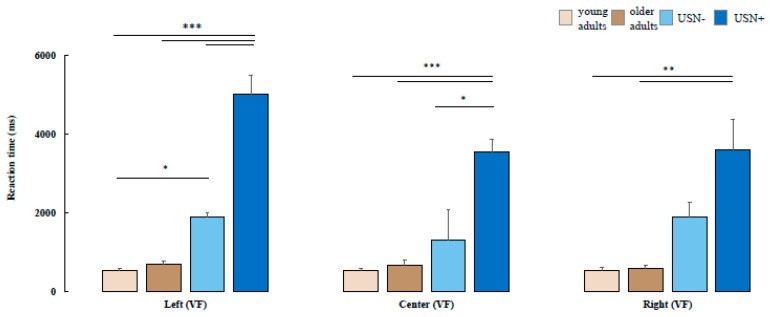
ET analysis showed a higher reaction time in the USN patients (USN+) with respect to stroke patients without USN (USN−) in the left and center visual field (VF), and with respect to healthy controls in each subdivision of the visual field of Task 1. * *p* < 0.05; ** *p* < 0.01; *** *p* < 0.001.

In the mixed-model ANOVA conducted on Task 1, comparing the sources of stimuli relative to the visual field (VF: left, center, and right) and group factors (group: young adults, older adults, USN−, and USN+), a statistically significant interaction was observed (VF vs. group) [F(2,6) = 2.5, *p* = 0.04]. Post hoc analysis revealed that the USN+ group exhibited increased response times to stimuli in each visual field source compared to the healthy control group. When comparing the USN+ group with the USN− group, a statistically significant difference was observed in the left and right visual field results. Interestingly, significant differences were also found between healthy young adults and USN− patients in the reaction time for the left visual field (see Figure 4) [34].

## 5. Discussion

The present study reports the preliminary testing and usability assessment of a single session of an immersive virtual reality (iVR) protocol to evaluate acceptability and eye-tracking (ET) differences between healthy controls and stroke patients with and without unilateral spatial neglect (USN). Additionally, a proposal is made for a triple-blind randomized controlled trial aimed at assessing the effectiveness of iVR training on visual perceptions in patients with USN. Our results suggest that iVR with ET biofeedback may be a suitable approach for addressing USN. The differences observed in the ET task across the four groups support the efficiency of the system in detecting ocular movement and the ability to locate and fixate on visual stimuli presented in the iVR scenarios, thereby providing a robust biofeedback marker. During the usability testing, no serious adverse events (AEs) were reported, and all participants tolerated the entire procedure well. Regarding minor AEs, neck stiffness was reported in a single case, which resolved spontaneously without the need for pharmacological intervention or other treatments. The investigation into the usability of the system yielded positive results among healthy young and older adult controls, as well as in stroke patients without unilateral spatial neglect (USN). However, in stroke patients with USN, usability was lower compared to the other sub-groups and did not reach statistical significance. The lower usability scores observed in patients with unilateral spatial neglect (USN) may be attributed to discomfort experienced during task execution. Interestingly, in the entire sample, the level of education emerged as a factor influencing the perception of usability of the immersive virtual reality (iVR) system. Notably, age did not negatively impact the usability perception of iVR, as noted by Lorenz and colleagues (2023), who found a correlation between age and System Usability Scale (SUS) results regarding iVR [35].

The ET results confirmed that patients with USN exhibited decreased performance compared to healthy controls and stroke patients without USN, particularly in the left visual field (VF). The diminished ability to find and fixate on a static target in the left VF is a characteristic symptom of USN, indicating visual perception deficits. Furthermore, differences were also noted in stroke patients without USN compared to controls in Task 1, albeit with better performance than patients with USN. This suggests that patients without USN also faced challenges compared to healthy controls in performing iVR tasks [34]. These results underscore the effectiveness of ET in providing real-time biofeedback, which can be instrumental in enhancing stimulation of the central nervous system [36]. Utilizing biofeedback through exercise gaming, virtual reality (VR), and immersive virtual reality (iVR) presents promising avenues for providing patients with immediate feedback to aid in exercise performance improvement [37,38]. This aspect is particularly crucial in unilateral spatial neglect (USN), where ET biofeedback pertaining to visual reach is closely linked to visual perception. By leveraging ET biofeedback, the exploration of neglected visual fields can be facilitated, thereby promoting recovery.

Furthermore, incorporating moving stimuli and environmental simulations in iVR training allows for the practice of USN functions in contexts that may not be feasible in a clinical setting (e.g., crossing a busy street). Additionally, ET results can serve as a tool for assessing visual exploration, offering an objective measure of USN recovery. The integration of instrumented evaluation of USN deficits alongside clinical assessment can enhance sensitivity during recovery or experimental observation.

In conclusion, the current findings support the utilization of ET biofeedback in an iVR protocol to investigate its potential for improving clinical outcomes in exploring the left visual field in patients with USN, through a triple-blind randomized clinical trial.

### Limitation

The current usability results were collected on a limited sample of patients and healthy controls in a single iVR session to test the feasibility of the planned iVR tasks. The exiguous sample size and the heterogeneity in the demographic characteristics limit the generalizability of our findings.

## 6. Conclusions

The usability and ET results of the present study suggest that the proposed iVR-ET biofeedback protocol is a safe and well-tolerated technique in patients with USN. The differences recorded in the ET results across healthy control and stroke patients with and without USN support the usability of ET biofeedback in the measure of VF exploration. Moreover, the real-time feedback can induce a performance response, supporting investigation into its use as a treatment approach.

## Figures and Tables

**Table 1:** Demographic characteristics.

Group (N)	Young Adults (6)	Older Adult (6)	USN− (4)	USN+ (3)
Age *	23.83 ± 1.72	48.16 ± 7.73	70 ± 8.16	56.66 ± 22.5
Gender	5 F, 1 M	4 F, 2 M	4 M	2 F, 1 M
Years of study *	16.57 ± 1.98	18.83 ± 3.31	14.24 ± 4.78	12 ± 6.55

Abbreviation: USN−, stroke patients without unilateral spatial neglect; USN+, stroke patients with diagnosis of unilateral spatial neglect. * Expressed as mean and standard deviation.

**Table 2:** ET and SUS results.

	One-Way ANOVA			Mixed Model ANOVA			
Group	Task 1	Task 2	Task 3	Task 1 (Left VF)	Task 1 (Center VF)	Task 1 (Right VF)	SUS
young adults	532 ± 147 *^b^	93 ± 7	3708 ± 983 *	530 ± 162 *^b^	524 ± 161 *	543 ± 143 *	83 ± 9
older adults	659 ± 205 *^b^	92 ± 9	3315 ± 813 *	700 ± 201 *	676 ± 297 *	600 ± 179 *	82 ± 12
USN−	1688 ± 832 ^ab^	80 ± 24	3981 ± 1076	1892 ± 982 ^ab^	1312 ± 660 ^a^	1894 ± 1559	74 ± 11
USN+	4077 ± 574 *^a^	52 ± 26	8020 ± 859 *	5013 ± 172 *^a^	3549 ± 1324 *^a^	3604 ± 657 *	53 ± 25
Post hoc analysis							
		Task 1	Task 2	Task 3	Task 1 (left VF)	Task 1 (center VF)	Task 1 (right VF)
young adults vs. older adults		0.961	0.999	0.988	1	1	1
young adults vs. USN−		0.006 ^c^	0.563	0.997	0.027 ^c^	1	0.901
older adults vs. USN−		0.014 ^c^	0.640	0.961	0.085	1	1
young adults vs. USN+		<0.001 ^c^	0.01 ^c^	0.050 ^c^	<0.001 ^c^	<0.001 ^c^	0.002 ^c^
older adults vs. USN+		<0 .001 ^c^	0.013 ^c^	0.030 ^c^	<0.001 ^c^	<0.001 ^c^	0.003 ^c^
USN− vs. USN+		<0.001 ^c^	0.139	0.101	<0.001 ^c^	0.013 ^c^	0.604

Abbreviation: USN+, stroke patient with unilateral spatial neglect; USN−, stroke patients without unilateral spatial neglect; data are expressed as mean ± standard deviation (Task 1 and 3 expressed in milliseconds and Task 2 in %). Significant results are reported in bold and flagged as follows: * *p* < 0.05 comparing USN+ with healthy controls, ^a^ *p* < 0.05 comparing USN+ with USN−, ^b^ *p* < 0.05 comparing stroke with healthy controls, ^c^ *p* < 0.05 at the post hoc analysis.

## Data Availability

The data that support the findings of this study are available from the corresponding author, GM, upon reasonable request. The data are not publicly available due to sensitive information contained.
